# Addressing Mild Cognitive Impairment and Boosting Wellness for the Elderly through Personalized Remote Monitoring

**DOI:** 10.3390/healthcare10071214

**Published:** 2022-06-29

**Authors:** Marilena Ianculescu, Elena-Anca Paraschiv, Adriana Alexandru

**Affiliations:** 1 National Institute for Research and Development in Informatics, 011455 Bucharest, Romania; adriana.alexandru@ici.ro; 2 Doctoral School of Automatic Control and Computers, University Politehnica of Bucharest, 060042 Bucharest, Romania; 3 Doctoral School of Electronics, Telecommunications and Information Technology, University Politehnica of Bucharest, 060042 Bucharest, Romania; 4 Faculty of Electrical Engineering, Electronics and Information Technology, Valahia University of Targoviste, 130004 Targoviste, Romania

**Keywords:** remote healthcare monitoring, MCI, smart environment, IoT, artificial intelligence, machine learning, deep learning

## Abstract

Mild cognitive impairment (MCI) may occur with old age and is associated with increased cognitive deterioration compared to what is normal. This may affect the person’s quality of life, health, and independence. In this ageing worldwide context, early diagnosis and personalized assistance for MCI therefore become crucial. This paper makes two important contributions: (1) a system (RO-SmartAgeing) to address MCI, which was developed for Romania; and (2) a set of criteria for evaluating its impact on remote health monitoring. The system aims to provide customized non-invasive remote monitoring, health assessment, and assistance for the elderly within a smart environment set up in their homes. Moreover, it includes multivariate AI-based predictive models that can detect the onset of MCI and its development towards dementia. It was built iteratively, following literature reviews and consultations with health specialists, and it is currently being tested in a simulated home environment. While its main strength is the potential to detect MCI early and follow its evolution, RO-SmartAgeing also supports elderly people in living independently, and it is safe, comfortable, low cost, and privacy protected. Moreover, it can be used by healthcare institutions to continuously monitor a patient’s vital signs, position, and activities, and to deliver reminders and alarms.

## 1. Introduction

People worldwide are expected to live longer, with a predicted increase of the population that is 60+ from 1 billion in 2020 to 1.4 billion in 2030 [1]. To tackle the resulting challenges, the European Commission (EC) is taking proactive measures, prioritizing initiatives that will contribute to building a healthy and active population for the future. Its projects and programmes focus on establishing a favourable environment that supports active aging, access to information technologies, and prolonging active working life.

Dementia due to Alzheimer’s disease (AD) is the most common malady of elderly people and a major cause of death all over the world, with an estimated outliving period varying from 3 to 8 years, depending on the person’s age [2].

Mild cognitive impairment (MCI) is an intermediate phase between normal cognitive decline caused by aging and AD. It involves mild cognitive changes that can be related to memory, thinking, judgment, and language, while the patient’s independence is not affected, and so it is difficult to distinguish between MCI and age-related cognitive decline [3]. However, in MCI, if treatment is applied to this degenerative process quickly, the injuries of the central nervous system can be limited, without leading to more serious and permanent damages, stopping deterioration and preventing subsequent disability. A smart environment that unobtrusively tracks daily living activities and the use of emerging technologies can help enhance elderly people’s cognitive wellness [4]. Smart environment technologies can perform remote monitoring and support individuals with MCI in living independently [5]. This approach enables persons with MCI to live in their homes when they need professional care, rather than in a medical institution. In this context, the main cons of a smart environment consist in providing an optimum remote configuration and security threats. 

The devices, technology, and services offered by the Internet of things (IoT) have facilitated the development of smart environments that could impact the life and cognitive behavior of the elderly. Remotely monitoring elderly people’s health and activities using IoT not only helps them live an independent life, but also decreases costs and response times in treating them. The main cons of IoT-based health monitoring consist of identifying the most appropriate device (considering the current cognitive condition of the patient with MCI), security threats, and the costs.

As the importance of early diagnosis of MCI has become known, work has been conducted on using artificial intelligence (AI) models based on machine learning (ML) and deep learning (DL) methods to predict MCI early and thus minimize the cognitive and behavioral deterioration. These ML and DL approaches have been found to detect subtle changes and disparities that are generally difficult to identify. The main cons of developing predictive models for MCI are the ethical issues, the challenge of obtaining reliable access to appropriate data, and that it is time consuming. 

This paper illustrates how RO-SmartAgeing, a system for remote monitoring developed inside the underdevelopment Romanian research project “*Non-invasive monitoring and assessment of the health of older people in a smart environment” (RO-SmartAgeing*), is designed to monitor, assess, and predict the health of patients with MCI. In a smart environment, the system integrates non-invasive IoT-based devices, for monitoring of health-related parameters; motion sensors, for collecting medical and lifestyle-related parameters; and a cloud platform for data storage, advanced data analysis, and AI-based disease prediction and presentation of results.

The RO-SmartAgeing system uses methods based on multivariate AI-based disease predictive models that integrate high-dimensional data. The results can be used for the identification of MCI individuals who might progress to AD within several years. Some of the measured vital parameters have the potential to be assimilated into (bio)markers as the most predictive for AD.

This paper aims to present how this system has the potential to fill some gaps from the Romanian research in the field, covering some aspects that are summarized as follows:

1. The RO-SmartAgeing system, due to its personalized smart environment, provides safe, minimally intrusive, low-cost, and privacy-protected remote monitoring with a strong accent on preventative and proactive functionalities.
2. The results obtained during the development of the system demonstrate that IoT-based devices selected so as to provide full and free post-purchase access to data collected during monitoring increase the potential of developing comprehensive applications targeted to better sustain the health care management of the elderly with MCI.
3. In Romania, there are currently no information systems that provide personalized remote health monitoring and interactive care of the elderly with MCI in an intelligent environment, customized according to the evolving needs, requirements, and dysfunctionalities of aged people, and that forecast the health status and MCI evolution by using modelling and AI-based prediction.

In this paper, related work is presented in Section 2. The system is described, and methods are discussed in Section 3. The results are presented in Section 4. A proposed evaluation criteria is provided in Section 5. A discussion is presented in Section 6. Finally, conclusions are drawn in Section 7.

## 2. Related Work

### 2.1. Enhanced Cognitive Wellness through Emerging Technologies

Several accessible innovative methods and tools for collecting, measuring, and early detection of behavioral activity patterns associated with MCI exist. They automatically evaluate the differences between adults with a normal age-related cognitive decline and those who are cognitively impaired by conducting ability tests during the execution of instrumental activities of daily living (IADL) [6], continuously monitoring low-level behavioral markers (steps taken, walking speed) in the home by using motion sensors and contact sensors [7], applying AI methods on data collected in sensor-rich environments to assess cognitive health status in the presence of a fixed set of predefined activities [8], using supervised learning approaches [9], and using other learning methods. Some existing recognition methods for different types of activities are presented in Table 1 [10].

This paper illustrates how the RO-SmartAgeing system, like the above-mentioned methods, collects data from wearable sensors. It also uses ML to recognize activity instances for fall detection based on gait events. It is provided with trigger alert capabilities in case of an abnormal value of a measured health parameter. The RO-SmartAgeing system additionally fills some of the gaps of these other methods: it clearly identifies the current location of the patient and the environmental conditions (see Section 4.1); it provides the capability of detecting abnormal behaviors based on health and daily activity monitoring over several months (see Section 4.2); it is based on data collected from the smart environment (see Section 4.1 and Section 4.2).

### 2.2. Smart Environments Augmented by AI

#### 2.2.1. Smart Environment

A sustained monitoring of the individual’s daily activities and health status could expand the detection and prediction of MCI symptoms and, consequently, increase the applied potential for better preventative treatments. A smart environment has augmented capabilities, being equipped with interconnected wearable or non-wearable devices integrated in its infrastructure with the role of observing and monitoring the everyday life of its inhabitant and providing proactive services aimed at improving their quality of life. The devices and sensors from a smart environment are designed to detect and monitor biomedical parameters (e.g., heart rate, oxygen saturation, blood glucose) [19], mobility and movement [20], daily activities [21], urinary-related infection problems [22], falling detection [23], and so on.

Several studies have proven the advantages of non-invasively monitoring elderly patients’ daily activities for the early detection of any cognitive decline or behavioral changes. Alberdi et al. [24] evaluated the detection of multimodal symptoms that influence the development of AD. They correlated characteristics of relevant activity with cognitive- or behavioral-related indicators in order to detect and provide results on mobility, cognitive relations, or depression symptoms. Once established, the symptoms can be predicted from monitoring daily activities and smart home data. In another study by Schinle et al. [25], automatic health monitoring personalization was considered using only a few ambient sensors to identify relevant indicators for AD detection. For that, they considered the patient’s wake up or bed-time hours, as well as their activities during night, considering their direct association with the onset of AD.

Like the above papers, the RO-SmartAgeing system also takes into consideration health parameter data when monitoring the development of MCI, which significantly helps with MCI prediction. In addition, it aims to correlate daily activity information not only with behavioral changes, but also with longitudinal health data, in order to observe any major changes in health.

#### 2.2.2. Continuum of Care Sustained by AI and Predictive Models

The continuous increase in the number of people suffering from MCI has spurred interest in the development of AI models and applications mostly concerned with brain magnetic resonance imaging (MRI) [26] and electroencephalography (EEG) [27] for early prediction of MCI. However, several studies have focused on identifying measures or correlations of measures for MCI prediction based on the patient’s medical data, including general activities or medical history. 

An automatic classification of healthy individuals and MCI patients was applied in the paper by Akl et al. [28]. They proposed an automatic detection of MCI in older adults via continuous home-based healthcare monitoring. In the training phase, each subject’s room activity distribution was estimated, and affinity propagation and relabelling using k-means were applied in order to cluster the activity distributions and to provide exemplar models for distinct clusters. The testing phase consisted of comparing the room activity distribution of each subject whose cognitive status was unknown with the exemplar models to assign the label of the exemplars that derive from the smallest normalized Kullbak–Leiber divergence. The labels were further applied to establish the subject’s cognitive condition. 

Ahamed et al. [29] used a public dataset with different types of daily activities of older adults with MCI along with their cognitive responses in order to detect any cognitive anomalies of the elderly and develop a model focused on detecting people with dementia. As pre-processing the data is necessary, a feature extraction step was performed, providing 59 extracted features that were then analysed in order to differentiate the data into the correct classes: cognitively impaired and healthy groups. However, the dataset proved to be imbalanced in the amount of data for the two categories, and a synthetic minority oversampling technique (SMOTE) process was applied to increase the record of the minority class, balancing the dataset. Several ML algorithms were used, and their performances were evaluated with k-fold cross-validation. The best performance for the balanced dataset was obtained using decision tree model with an F-score of 90.74%, but the random undersampling boosting (RUSBoosted) ensemble model obtained good F-score results with both balanced and imbalanced datasets: 87.38%, and 88.89%, respectively. 

Predicting the first signs of dementia, a more severe stage of MCI, has been analysed in several studies, including [30]. Arifoglu et al., focused on sensor-based activity recognition and signs of abnormal behavior in older adults suffering from dementia. Considering the fact that collecting actual data is a difficult task, they generated synthetic data associated with behavioral features of people with dementia. Consequently, they have considered convolutional neural networks (CNNs) for activity patterns and abnormal behavior detection, activity recognition being an essential problem of sequence labelling, resulting in highlighting of the abnormal behavior as it represents a deviation from the normal pattern. The applied CNNs have focused on three types of architectures: 1D convolutional, on the temporal dimension; 2D convolution, on the temporal and feature dimensions; and 2D with a long short-term memory (LSTM) layer-based recurrent neural network (RNN). The value of the sensitivity of LSTM-based CNN was computed as 98.67% and for 2D CNN as 85.33%, which outperformed the state-of the-art results (using naïve-Bayes, hidden Markov models, etc.).

To predict the progression of MCI in older adults, Narasimhan et al. [31] applied the RNN-LSTM model on activity data acquired from sensors and the subject’s health data recorded at different moments. The authors have focused on leveraging the temporal evolution of MCI in order to improve its future prediction according to its development in time. That is why the model used in this paper is based on RNNs, as they have shown good performances in terms of longitudinal data. Therefore, the applied method uses LSTM RNN, highlighting the importance of this type of neural network in the healthcare domain. An initial experiment resulted in an average testing accuracy of 77.5% using a synthetic dataset based on real-world cognitive measures.

As MCI impairments progress over time, remote healthcare monitoring applications enhanced by AI prediction models for this condition can recognize certain temporal patterns; hence, they can allow the possibility of early MCI detection. 

The RO-SmartAgeing system is able not only to collect the relevant monitored elderly person’s lifestyle data but also to use public datasets for the development of AI algorithms based on health data and activities recognition. These AI models can be applied on larger datasets with relevant features in order to be carefully developed for MCI detection. In addition, they can be concatenated with the health parameters in order to correlate the development of MCI with its potential progression.

### 2.3. Patient-Centric Remote Monitoring

The value of the global digital health market was estimated at $96.5 billion in 2020, with a 15.1% estimated increase in the compound annual growth rate (between 2021 and 2028) due to growing investments in digital infrastructure and continuous software upgrades [32].

Remote health monitoring systems are increasingly becoming a necessary solution for the provision of personalized healthcare services, being less intrusive and closer to the particularities and needs of the patient. A remote health monitoring system includes minimal devices (including smart ones), IoT-based networks, and wireless sensors for real-time collection, storage, and analysis of various medical parameters. IoT technology is an important factor that can support a wide range of capabilities of these systems, including access to a huge amount of various health data. Industry 5.0 and 5G telecommunication technology led to the development of cost-effective sensors and devices for real-time remote monitoring and capturing of the data [33]. In the meantime, the costs associated with healthcare services have been decreasing.

*ICT 4 life—ICT services for Life Improvement for the Elderly* [34] is focused on real medical problems of people with dementia, Alzheimer’s, or Parkinson’s disease. It involves advanced multisensory-based analytics and integration with biomedical devices for gathering patient activity and health status information.

The development of the RO-SmartAgeing system, especially in the current context of the COVID-19 pandemic, was adapted in order to allow tailored patient-centric remote monitoring.

## 3. System Description and Methods

### 3.1. Main Objective

The main objective of *RO-SmartAgeing* is the development of a system for monitoring, assessing, and predicting the health of patients with MCI, which collects health and motion data from IoT sensors and devices, transmits it, and stores it on a cloud platform for further data analysis applications.

The RO-SmartAgeing system relies on:

- Monitoring health and ambient parameters, daily movement, activities behavior, and routine tasks through accurate gathering of a quite comprehensive range of data collected by IoT devices (Withings devices, depth cameras, and devices built inside a project (the so-called “Blackboxes” and the “Leg Band”) for health parameter monitoring—such as heart rate, electrocardiogram (ECG), blood pressure, sleep-associated parameters, body position, bioelectric impedance analysis, body fat, total body water, muscle mass, bone mass—and motion and environmental parameters);
- Using open data sources for MCI.

Data obtained from standard cognitive tests (as Mini-Mental Test Examination (MMSE), Alzheimer’s Disease Assessment Scale-Cognitive (ADAS-Cog)) are used to form intelligent models to detect the onset of MCI. All the obtained data are stored into a database and analysed in the cloud using AI and ML techniques for identifying patterns in cognitive state. One of the main goals was to develop multivariate AI-based disease predictive models that integrate the previously mentioned high-dimensional data for identification of MCI individuals who progress to AD. 

The main targeted beneficiaries of the RO-SmartAgeing system are the elderly, including those with MCI, their current healthcare specialists, and caretakers/family.

### 3.2. Method

**Figure 1:**
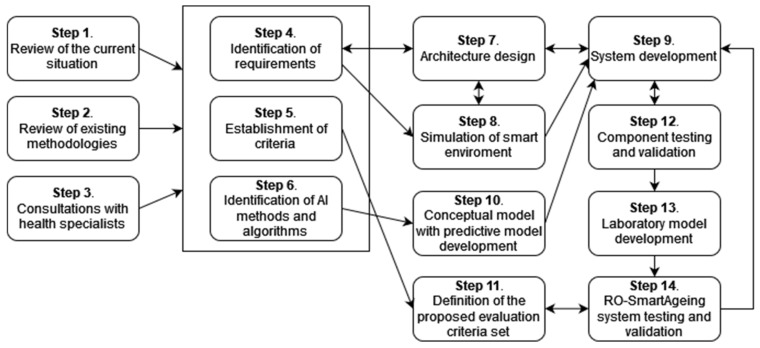
Steps for implementing the RO-SmartAgeing system.

In order to implement the RO-SmartAgeing system, the following steps have been followed:

- Step 1. Authorized scientific sources. Performing a systematic review of a large number of authorized scientific sources in order to clearly identify the most relevant information about the current situation (with an emphasis on the Romanian one) in the domains of population ageing; healthcare services for elderly patients, including those with MCI; current and emerging digital technologies; and healthcare solutions;
- Step 2. Review of existing methodologies. Performing a literature review to identify currently used methodologies for the assessment of digital systems and technology, as well as the current legal framework and guidance;
- Step 3. Consultations with health specialists. Organizing several rounds of consultations with health specialists; taking into consideration the specificities in the Romanian health, social, and IT domains, and the most significant drawbacks, limitations, deficiencies, and facilities;
- Step 4. Identification of requirements. Identifying the functional and non-technical requirements for the digital system, based on the above-mentioned research;
- Step 5. Establishment of criteria. Setting up criteria for selecting the most appropriate devices and technologies according to the special needs and demands of elderly people with MCI and their physicians, for the development of an efficient, tailored smart environment;
- Step 6. Identification of AI methods and algorithms. Performing research for identifying high-performing AI (DL and ML) methods and algorithms, as well as MCI-related open data, used in the development of predictive models;
- Step 7. Architecture designing. Designing RO-SmartAgeing system based on an IoT- and microservice-based, multilevel architecture in order to provide a high degree of flexibility and scalability;
- Step 8. Simulation of the smart environment. Creating a smart environment in a laboratory setting that reproduces as closely as possible the real living conditions of an elderly person;
- Step 9. System development. Developing the system using an iterative approach;
- Step 10. Conceptual model with predictive model development. Including the predictive models in a conceptual model conceived to concatenate the health parameters collected via RO-SmartAgeing smart environment and the MCI-related data from the open data sources, in order to obtain a more accurate and trained result;
- Step 11. Definition of the proposed evaluation criteria set. Defining a proposed evaluation criteria set by taking into consideration the high-rate dynamics of the advances in the field of remote health-monitoring solutions;
- Step 12. Component testing and validation. Testing each component of the system and validating it in the laboratory environment before its integration into a comprehensive system;
- Step 13. Laboratory model development. Developing a laboratory model of the RO-SmartAgeing system;
- Step 14. Testing and validating the RO-SmartAgeing system. Testing and validating the RO-SmartAgeing system in laboratory and real conditions (Figure 1).

### 3.3. System Architecture

The smart environment comprises IoT-based devices for measuring vital signs, health, motion, and ambient parameters, and depth cameras for monitoring activities. The gathered health data is analysed and processed in edge, fog, and cloud computing, and afterwards, it is used in different queries, reports, and applications that support different healthcare services. 

**Figure 2:**
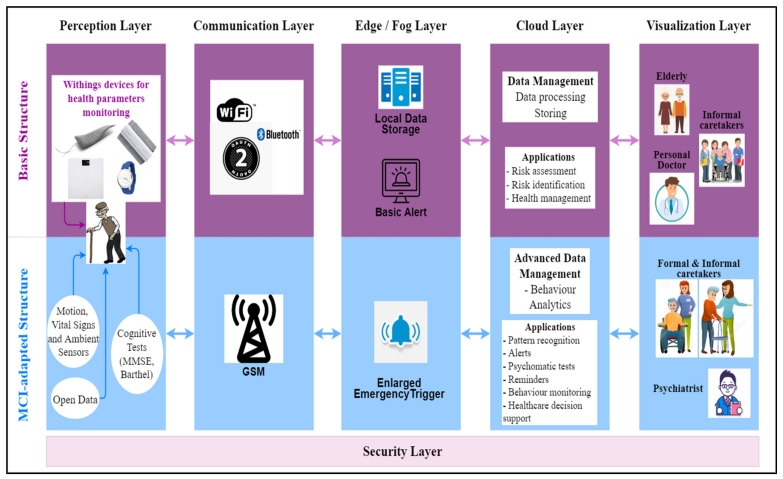
The functional architecture of the RO-SmartAgeing system with highlighted MCI-related aspects.

The system functional architecture is structured into six layers (Figure 2): Perception, Communication, Edge/Fog, Cloud, Visualization, and Security.

- The *Perception Layer* consists of a variety of devices and other type of sources for collecting health-related data;
- The *Communication Layer* allows the raw data to be transferred to the next layers;
- The *Edge/Fog Layer* performs preliminary analytics in order to analyse the data and trigger alarms, in real time (basic alerts). An enlarged emergency trigger functionality, built around some specificities of MCI, is provided for a fast response. Data is temporary stored in a local data storage;
- The *Cloud Layer* performs long-term analytics and data management in order to generate specific health-supporting applications, including the ones directly related to MCI, such as personalized predictive models;
- The *Vizualization Layer* gives access to the applications that provide the system users with different visualizations (in different formats or reports) of the stored data or new insights obtained from advanced processing;
- The *Security Layer*—the privacy, confidentiality and security issues associated to health data and a digital system are addressed across all layers, according to the local specificities and threats.

## 4. Implementation of RO-SmartAgeing System

### 4.1. Tailored Smart Environment for MCI Patients

The smart environment, as a main part of the RO-SmartAgeing system, comprises IoT-based Withings devices for health parameters monitoring, as well as devices able to monitor and track health parameters and human activity recognition, which represent essential measurements for MCI prediction. The smart environment is managed through two of the main components of the system, namely the Medical and Monitoring components. 

The associated database of the system contains broad medical history information, as well as continuously or periodically collected data, which can be further analysed and submitted for advanced data management, leading to the following applications: behavior monitoring, trigger alerts, reminders, AI (DL or ML)-based prediction, health decision support, and other specific applications directly related to MCI. Besides these non-intrusive technologies that operate independently of the monitored elderly person, some interactive applications, such as psychometric tests, will be also designed to complete the elderly person’s profile. 

There are four Withings devices: (1) MoveECG smartwatch; (2) blood pressure monitor; (3) smart scale; (4) smart sleep analyser.

Three monitoring devices have been designed, projected, and developed within the project (Supplementary Section S1 presents the technical schemas—which include the applied sensors—and the final kit for the developed monitoring devices):

- The Medical Blackbox is responsible for monitoring the healthcare parameters. It has been designed to offer the possibility to manage personalized monitoring according to the patient’s needs, as it is based on accessible, independent, and Arduino-compatible sensors.
- The Ambient Blackbox targets the measurement of the home-based environmental parameters while the patient is normally practicing their daily activities.
- The Leg Band includes an accelerometer and gyroscope-based sensor for patient’s movements, positioning, and fall detection, these measurements being essential for an MCI-related condition.

The data from these devices is further transmitted, via the Node MCU, to the Raspberry Pi gateway, and consequently to the ICIPRO Cloud platform in order to be visualized and processed.

The devices comprising the RO-SmartAgeing system and their corresponding targeted measured parameters and technical characteristics are (see Supplementary Section S2 for more details):

- *Medical Blackbox*—for pulse and oxygen in blood; body temperature; volatile organic compounds from breath; urine biochemistry;
- *Leg Band*—for fall detection and number of steps;
- *Ambient Blackbox*—for ambient temperature; environmental temperature and humidity; ambient light; flammable gases and smoke; infrared signal caused by motion; pressure and altitude; air quality;
- *Smart depth camera (Orbbec Persee)*—human activity recognition [35];
- *Withings Smartwatch*—ECG; pulse; GPS; activity monitoring; score for sleep quality assessment; sleep duration; sleep cycles (deep, light, REM sleep) [36];
- *Withings Blood Pressure Monitor (BPM)*—blood pressure; ECG; pulse [37];
- *Withings Sleep monitor*—score for sleep quality assessment; breathing disorders; sleep duration; sleep onset and waking time; sleep cycles: deep, light, REM; pulse during sleep; average pulse; snoring duration [38];
- *Withings Smart Scale*—body position detection; bioelectric impedance analysis; body fat (%); total body water (%); muscle mass (kg or lb.); bone mass (kg or lb) [39].

Moreover, the Withings devices provide not only the advantage of easily visualising the measured data on a smartphone, but also of collecting all these data in the platform of the RO-SmartAgeing system, based on the Withings API.

Through the Monitoring component, the IoT-based devices are managed in order to ensure a safe and efficient process from the collecting of measured data to its storage in the RO-SmartAgeing database. As the RO-SmartAgeing system can be personalized, there are no restrictions regarding the localization in the room of the Ambient Blackbox or for the other devices for health parameters. The smart depth camera should be mounted near the ceiling so as to ensure the widest field of view. The devices were chosen in such a way as to avoid any interference between them and with no other specific requirements. The data are locally visualized and a simple Wi-Fi connection is necessary for its transmission to the cloud.

Considering the negative impact MCI might have on the risk of further development of dementia, the RO-SmartAgeing system can target the longitudinal monitoring of the patient’s decline and their cognitive behavior in order to detect any observable signs that can impact the development of this condition [10].

For the MCI monitoring, RO-SmartAgeing includes a smart depth camera (Orbbec Persee) to track the patient’s activity and their way of interacting with the environment in order to observe relevant changes in their behavior. In addition to this, the sleep tracking mat used to analyse the sleep activity of the targeted patient provides an essential monitoring for an MCI individual, as the patient’s wake up time, bed time, and night time are relevant parameters for this condition [40]. Considering that hypertension is also a relevant parameter in MCI development, a blood pressure monitor is being used to measure the blood pressure in order to keep it among the reference values. A smartwatch is also included in the system as it can easily measure significant parameters, such as ECG, pulse, movements or activities, number of steps, or GPS monitoring. As it does not provide any inconvenience in wearing it, the MCI patient can be easily monitored with it. Similarly, the leg band, which consists of a six-axis accelerometer and gyroscope, is also included for MCI monitoring in order to observe a patient’s possible fall or decline. 

### 4.2. Predictive Models for MCI—Conceptual Approaches

Over time, multiple studies have examined the influence of different types of data on MCI: hear-disease-related data [41], demographic information [42], daily activities [43], or kinetic data [44].

Joining the RO-SmartAgeing system with a potential AI can provide to the development of MCI prediction applications. The obtained impact on early detection of this type of condition or other related signs is of great interest. Two conceptual models have been proposed in order to enhance the capabilities of the RO-SmartAgeing system for early classification and detection of MCI-related conditions: heart diseases or falling-based kinetic data. These two models based on open-source data will be further integrated with the data collected through the RO-SmartAgeing system in order to provide it with a predictive model able to detect the early signs of MCI.

**Figure 3:**
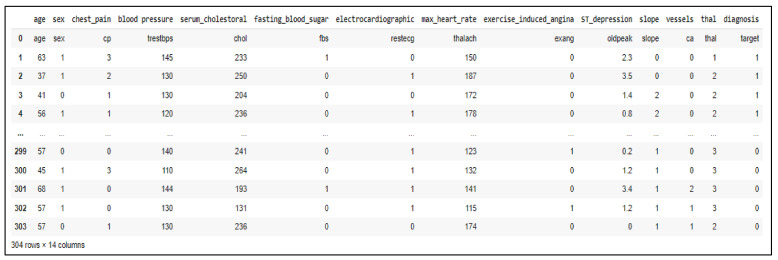
The UCI Heart Disease dataset.

The first conceptual approach was described in Paraschiv et.al. [45], and it is based on *the classification of heart diseases appearance*, which is a common factor influencing the development of MCIMCI. The proposed method is an ML- and DL-based conceptual model that uses an open-source dataset, the UCI Heart Disease Set [46], which includes physiological and biological parameters such as age, chest pain type, resting blood pressure, resting ECG results, and maximum heart rate, which are essential for monitoring an MCI patient. In addition to this, the dataset also contains the following: serum cholesterol, fasting blood sugar, exercise-induced angina, ST segment difference between resting ECG and exercise-based ECG, the slope of the exercised ST segment peak, and the number of major vessels colored by fluoroscopy and thalassemia (which is a hereditary blood disorder). The target of this dataset is represented by a binary variable (1 = no heart disease, 0 = heart disease). The dataset is presented in Figure 3.

**Figure 4:**
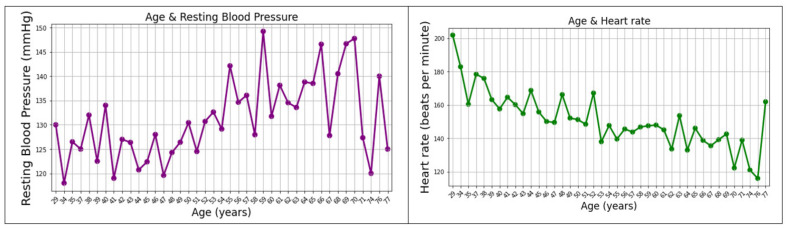
Age and resting blood pressure plot (**left**); age and heart rate plot (**right**).

Starting from [45], an analysis of the dataset has been applied, resulting in the following two plots in Figure 4. It is observed that the values for resting blood pressure rise with ageing, compared to the heart rate values, which have a decrease tendency; it is generally known that, over time, it might take longer for the heart rhythm to increase and, also, longer to diminish afterward.

**Figure 5:**
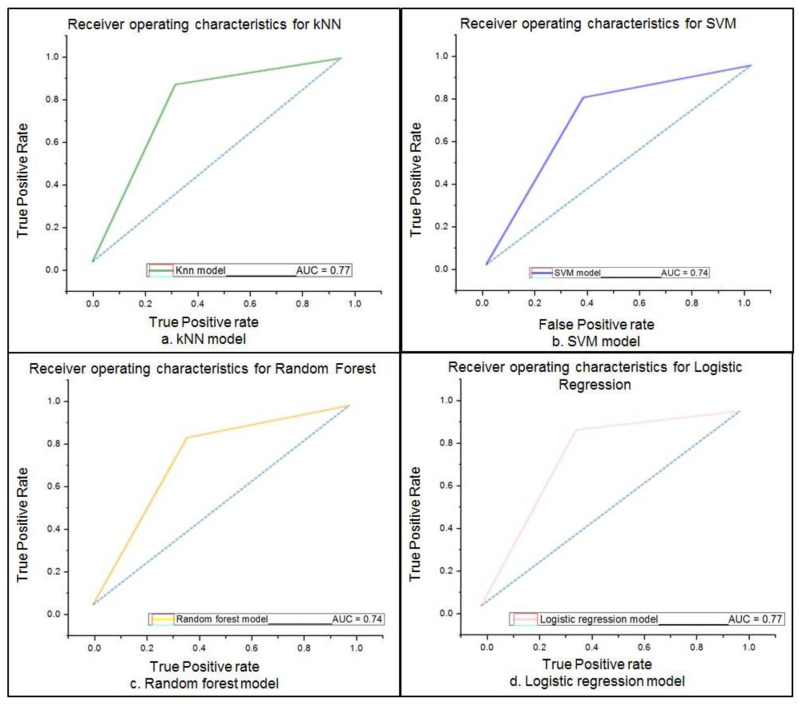
ROC curve for different ML models: a. kNN model; b. SVM model; c. Random forest model; d. Logistic regression model.

The proposed ML algorithms included k-nearest neighbour (kNN), support vector machines (SVM), random forest, and logistic regression models, as well as an optimized four-layer neural network for the DL method. The dataset for the ML algorithms was split into 80% for training and 20% separate data for testing. The metrics used for performance evaluation were represented by the following: *sensitivity*, *specificity*, *accuracy,* and *receiver operating characteristic (ROC) curve*. Among the well-known ML models, kNN is a supervised method that attempts to identify all the nearest neighbours around the new and unknown data point in order to determine which class it belongs to. Therefore, kNN computes the distance from all the data points located in the proximity of the unknown point and extracts only the points with the minimum distance. In order to apply kNN on the UCI Heart Disease Dataset, the number of neighbours (5) and Euclidean distance were selected for computing the algorithm, based on the following equation:

(1) $$Euclidean\;distance\left(x^{*},\;x\right)=\sqrt{\sum_{i}^{m}{\left(x_{i}^{*}-x_{i}\right)}^{2}}$$

where $x^{*}$ represents the feature vector of the unknown data point, and x is the feature vector of one data point in the training set. In addition, m is the number of features used for the prediction. The resulted evaluation metrics are as follows: *sensitivity = 87.09%, specificity = 66.67%,* and *accuracy = 76.01%.* The ROC curve is plotted in the following figure (Figure 5a).

Another model used for classification of the UCI Heart Disease dataset was SVM. The principle behind the SVM algorithm is based on plotting each data item as a point in n-dimensional space (n-number of features), each feature value being a coordinate value. The classification is performed by identifying the distinction between the hyperplane for the two classes. When applied to the dataset with a radial basis function (RBF) as kernel, the SVM model classified the dataset with the following evaluation metrics: *sensitivity = 83.87%, specificity = 63.34%,* and *accuracy = 74%.* The ROC curve for this model is illustrated below (Figure 5b).

Random forest is a widely used ML algorithm for classification and regression tasks. It is based on building ensembles of decision trees that provide votes that classify the data based on the relevant features. The model was applied on the dataset with 300 decision trees and *gini* as the function that measures the quality of trees splits. The gini function is generally used with ML algorithms as it computes the probability of a feature that is incorrectly classified when it is arbitrarily selected. Therefore, the obtained evaluation metrics are as follows: *sensitivity = 83.9%, specificity = 64%,* and *accuracy = 73.8%.* The ROC curve for the random forest model applied on the UCI Heart Disease dataset is illustrated in Figure 5c.

Logistic regression, another ML model used for the classification of the UCI Heart Disease dataset, is an algorithm used for classification tasks that is able to predict a categorical dependent variable using a set of independent variables. The best performance among the ML algorithms was obtained with the logistic regression with the following evaluation metrics: *sensitivity = 91%, specificity = 65%,* and *an accuracy = 78%*. The ROC curve is displayed in Figure 5d.

**Figure 6:**
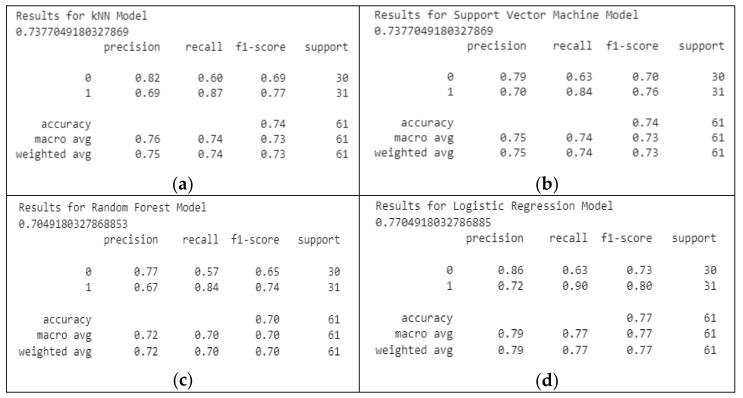
The classification reports for the ML algorithm: (**a**) classification report for kNN model; (**b**) classification report for SVM model; (**c**) classification report for random forest model; (**d**) classification report for logistic regression model.

The classification reports for the applied ML algorithms are presented in the following figure (Figure 6).

**Figure 7:**
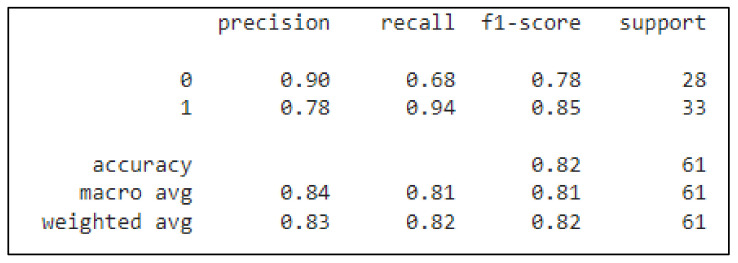
Classification report for the DL method.

Despite ML being used for classifying the UCI Heart Disease dataset, a four-layer CNN-based model was also applied to perform the classification task. The accuracy of the provided model obtained a score of 82%. The classification report for the model is provided in the following figure (Figure 7).

The classification report of the DL method shows the f1-score, precision, and recall of each class, and the overall testing accuracy, as this is an important step in performance evaluation.

A comparative table listing the performances of the ML and DL models is listed below (Table 2).

According to the classification reports and the performances table presented above, the random forest model obtained the lowest performance compared to the logistic regression model, which proved to be a reliable algorithm for this type of application. In addition to this, the DL model, based on only four hidden layers, managed to obtain better results. Additional upgrades for this model are foreseen.

The second conceptual approach is based on the development of a better *patient’s fall management* (Ianculescu et al. [47]). In this research, spatial and temporal characteristics (such as step length, step duration, step width), along with the kinetic features, were analysed based on the data collected from the leg band and smartwatch. In addition to this, images of body parts were also extracted from the smart depth camera videos in order to classify the data. The classification was performed by the ML-based SVM algorithm. 

The training was based on walking, standing, going up and down the stairs, and running, this data being recorded and validated with the leg band and smartwatch included in the smart environment of the RO-SmartAgeing system. The recorded actions were included in the database for improving the fall prevention feature. Afterwards, some tests were applied in order to visualise the accuracy of the implemented model. 

The model based on the acquired smartwatch data performed better on running gait (96% accuracy), whereas the SVM model based on data collected from the leg band and the camera performed best on the walking gait (98% and 100%, respectively). The wearable sensors are further used to detect falls, and the smart camera to validate them. This method provides an essential step towards the development of an application for the RO-SmartAgeing system that could detect the patient’s movements and trigger an alarm if a fall is detected. 

This could be a prerequisite for MCI prediction since fall detection is of critical importance for this condition. It is considered that the data from the sleep tracking mat would be a positive addition to the model in order to prevent any falls that could occur during the night. 

The previously described conceptual models are used, based on more open-source data and data gathered through the RO-SmartAgeing smart environment, in order to classify and evaluate it and to further apply on MCI patients. 

## 5. Results

### 5.1. Functionalities for Monitoring MCI Provided by RO-SmartAgeing System

The RO-SmartAgeing system offers support for preventative, proactive, and tailored health services for elderly people with MCI by using an accessible and age-friendly platform.

The functionalities provided by the RO-SmartAgeing system are designed and implemented so as to take into account the health and technical literacy levels of the user. Moreover, they are structured according to three main components, as follows:

- **The Medical Component**
  ◦ *Scope*: It allows personalized monitoring and dynamic management of medical data and information of an elderly patient with MCI. It allows the collection (in real time, at changeable established intervals) of data and information related to the evolution of the health status, the environment in which he/she carries out the daily activities, or other information essential to support the provision of a diagnosis or treatment.
  ◦ *Examples of provided functionalities*: Management of the trigger alerts; monitoring planning; management of collected data; health report generation; evaluation of collected data and information in the context of MCI; planning daily activities; personalized remote configuration of the smart environment; administration of the interactions among patient, caretaker/family, and healthcare specialists.
- **The Monitoring Component**
  ◦ *Scope*: It provides functionalities for the collection of data from the IoT-based devices that are integrated in the smart environment, and the transmission of this data to the Edge/Fog and Cloud Computing, where it is filtered, aggregated, analysed, and stored in the RO-SmartAgeing database. In the case of detecting an abnormal value of a measured parameter or an accidental fall, specific functionalities for alerting the caretaker and personal physician are triggered.
  ◦ *Examples of provided functionalities*: Management of monitoring biomedical parameters; management of monitoring ambient parameters; management of smart environments components; management of monitoring current activities; management of monitoring fall detection and movements; generating trigger alerts.
- **The Service Support Component**
  ◦ *Scope*: It provides specific information on a number of issues associated with supporting active, independent, and healthy ageing.
  ◦ *Examples of provided functionalities*: Recommendations for healthy and independent living for the elderly; elderly self-assessment questionnaires on the quality of life and well-being of the elderly; support information for caretakers regarding information on specifics of care for the elderly; services for supporting/facilitating social relationships and cognitive abilities of the elderly; (self-)assessment of cognitive ability; personal fall risk awareness assessment; fall prevention information management; management of digital technologies for the elderly; information on legislation and regulations for the elderly; management of COVID-19 disease.

**Figure 8:**
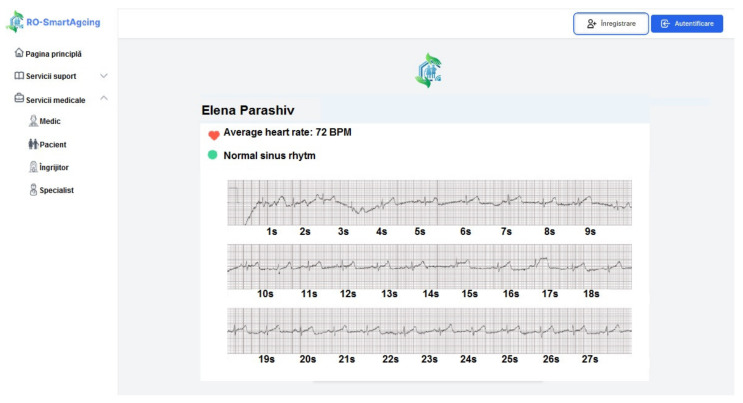
Measured ECG with a Withings device displayed on the RO-SmartAgeing platform.

Figure 8 illustrates how the ECG is displayed on the RO-SmartAgeing platform after it was measured with the Withings blood pressure monitor.

**Figure 9:**
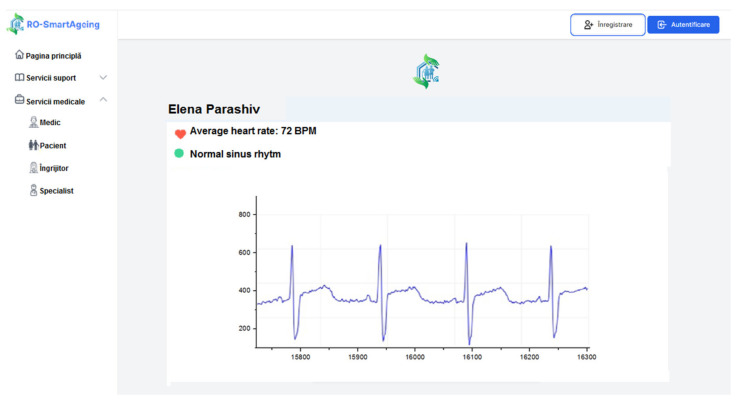
Measured ECG with the Medical Blackbox displayed on the RO-SmartAgeing platform.

Figure 9 illustrates how the ECG is displayed on the RO-SmartAgeing platform after it was measured with the Medical Blackbox.

**Figure 10:**
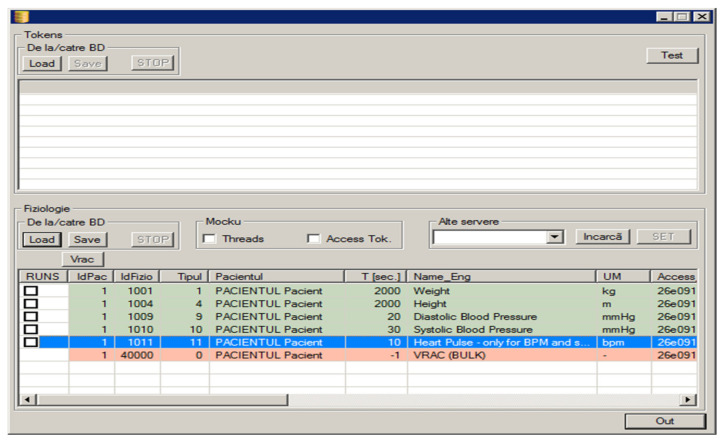
Illustration of the result of the application designed to obtain health parameter measurements for a certain patient performed with a set of Withings devices.

**Figure 11:**
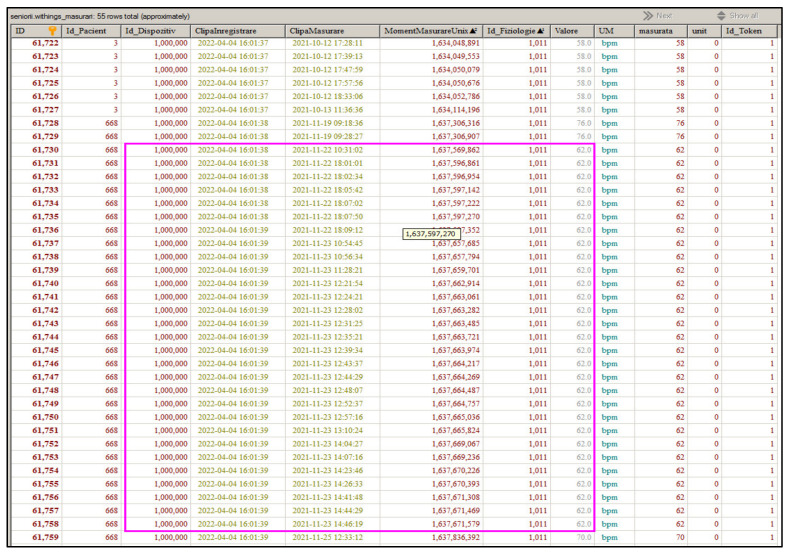
Illustration of recording of a series of consecutive measurements of a single parameter performed with a Withings device.

**Figure 12:**
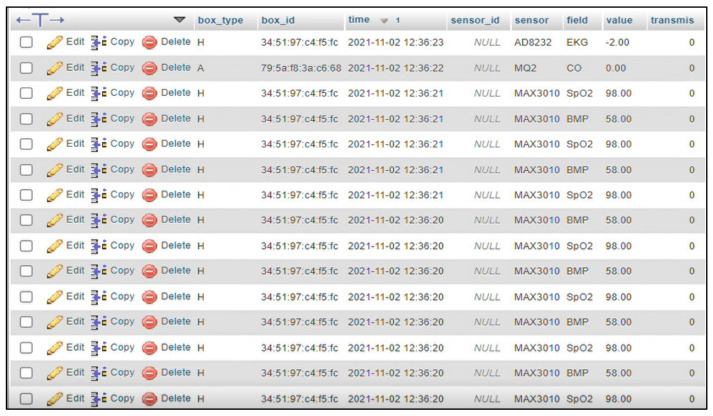
Illustration of the representation in the RO-SmartAgeing database of the measurements performed with a Withings device.

Another aspect that can be mentioned about the reliability of the functionalities provided by the RO-SmartAgeing system is related to the frequency with which the measurements are made and the time intervals at which the collected data is sent to the Cloud database. The measured data is strictly associated to the specific patient, device, and the time of measurement (Figure 10, Figure 11 and Figure 12).

Figure 10 presents the correlation between a certain patient and the necessary information associated to different health parameters that have to be recovered from the Withings Cloud.

Figure 11 presents how consecutive measurements of a certain parameter gathered through a Withings device are stored in the RO-SmartAgeing Cloud database.

In a first step, all the measured values are stored in the Withings Cloud; at an established interval, an automated request is sent from the RO-SmartAgeing system, and all the data is transmitted from the Withings Cloud. In the next step, the last moment in which data was loaded in the RO-SmartAgeing database is checked; that specific time is then identified in the new set of data. Only the data below that moment of time are analysed, processed, and stored in the RO-SmartAgeing database in order to avoid duplicates and redundancies. 

Figure 12 illustrates the result of a succession of measurements performed with a Medical Blackbox that comprises different sensors for different health parameters. All the relevant information is stored in the database.

All the data collected by the devices from the RO-SmartAgeing system is to be used by the developed functionalities of the system. At the same time, some of this data is suitable for training the above-mentioned predictive model for MCI. 

### 5.2. Proposed Evaluation Criteria for Health Remote Monitoring Systems for Elderly Patients, Including Those with MCI

Despite its huge negative impact on the health, social, and economic domains, both at the individual and society levels, the COVID-19 pandemic can be perceived as an accelerating driver for the development and deployment of remote health-monitoring solutions, due to the increased urgency to sustain healthcare services. Because of the lockdowns, restricted access to hospitals, and limited access to certain medical services, the elderly—one of the main vulnerable groups of population—have been directly affected from the point of view of access to qualitative, continuous, and efficient health services. Moreover, due to the pandemic, the access to mental health services was disrupted—as confirmed by 60% of the participants from 130 countries who responded to a 2020 World Health Organization survey [48]—even if the specificities of MCI impose a close relationship among the patient with MCI, their physicians, and their caretakers, together with an accurate tailored management of the disease. In this context, it becomes necessary to establish distinguishing *evaluation criteria* for remote health monitoring for elderly patients, including those with MCI, in order to: (1) provide a comprehensive assessment for digital health solutions with different levels of maturity, from the concept stage onwards; (2) identify good practices for implemented digital solutions that have proven performances, benefits, reliable outcomes, efficiency, equity, safety, usability, and accessibility. 

The most important arguments that sustain the need for specific evaluation criteria are as follows:

- A validated good practice can be more quickly replicated, adapted, and implemented;
- The stakeholders have the necessary information to be able to estimate if the functionalities and features of a good practice correspond to their need and objectives;
- The developers have clear targets for improving health monitoring according to the evaluation results to refer to;
- The evaluation criteria represent a solid base for an objective validation process of a digital solution in order to see if it may be undertaken as a good practice.

The design and development of the RO-SmartAgeing system have taken into consideration the specific needs and demands for both the main users (patients, health specialists, caretakers) and the legal entity that coordinates the system (healthcare organization, independent physician) in Romania. Therefore, the RO-SmartAgeing system was taken as a benchmark for defining a *specific evaluation criteria set for the remote health monitoring of elderly patients/patients with MCI* in Romania.

The defined criteria, in accordance with current *Health Technology Assessment* Guidelines for *Digital* Health Technologies [49,50,51,52,53,54,55] and adapted from [56], are also related with the IoT-based multilevel architecture of a remote health-monitoring system such as RO-SmartAgeing, that is, the *Perception Layer, Communication Layer, Edge/Fog Layer, Cloud Layer, Application, and Visualization Layer* (as it was presented in Figure 2).

The specific evaluation criteria set is structured into criteria and sub-criteria classified according to six thematic modules. The sub-criteria have been selected to provide in-depth information associated with a certain criterion.

The rationale for establishing the six modules (*General; Parameter Acquisition; Analysis; Application and Business; Technical Features; Available Evidence on Outcomes)* is based on the life cycle of a remote health-monitoring solution, the specific features, outputs, outcomes, and impact. The defined criteria are presented in Supplementary Section S3.

Each criterion and sub-criterion is defined in such a way as to have the following characteristics: clear and easy to understand, distinguishable, measurable, related to digital health for remote monitoring, comprehensible for its category, actual, and in accordance with current standards and terminology of health technology assessment for digital health technologies. As information and communications technology (ICT) is a highly dynamic field, digital solutions are emerging and upgrading fast. In this context, for an accurate and proper assessment, the maturity levels of remote health-monitoring systems for elderly patients, including those with MCI, were taken into consideration and defined as follows (adapted from [56]):

- Concept—system under development;
- Emerging—system during testing phase;
- Pilot—an experimental version of the system is tried out in a real environment;
- Implemented—a functional version is ready-to-market and used in healthcare units;
- Upgraded—optimized versions are available;
- Cross-border—the system is integrated and functional in medical practices from different countries.

Each maturity level is marked with a value between 1 and 10, 1 being the lowest rate.

The evaluation criteria set was defined for assessing an IoT-based remote healt-monitoring system, with a patient-centric approach. The specific criteria that target the MCI-monitoring capabilities are as follows: type of beneficiaries, beneficiary basic characteristics; all the criteria from Module 1: Parameter Acquisition, Module 2: Analysis, and Module 5: Available Evidence on Outcomes; patient profile; health status management, impact on beneficiaries, most used technology, content quality, usability, accessibility, privacy and security and confidentiality, and characteristics of the life cycle of the solution. These criteria assess the direct relationship among the patients with MCI, their physicians and caretakers, the degree to which the system is integrated in the patients’ life, and the outcomes in terms of quality of life, medical services, and health status.

### 5.3. Organizing the Testing of RO-SmartAgeing System

Taking into consideration the sensitive nature of health and personal data, a result-oriented testing plan needed to be elaborate, considering multiple aspects of this digital health solution: functional and non-functional requirements, software, components of the smart environment and other used technologies, different types of users/beneficiaries, and provided functionalities. At the same time, the usability, accessibility, acceptability, and age-friendliness features are taken into consideration.

**Figure 13:**
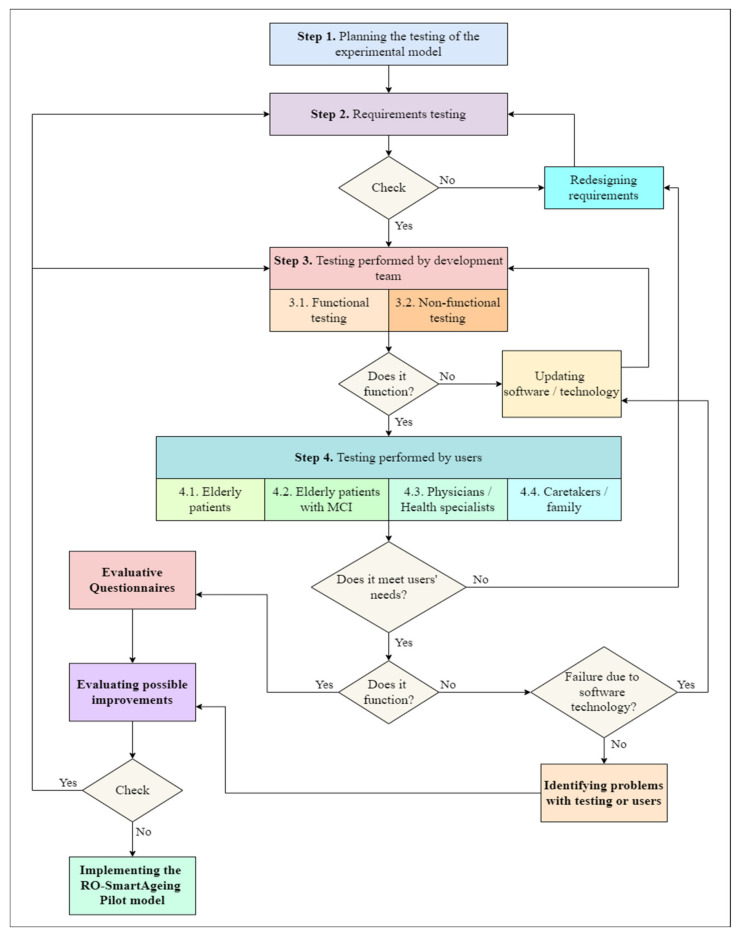
Algorithm for organizing the testing process of the RO-SmartAgeing system.

In this respect, the organization of the testing process was elaborated as follows (Figure 13):

- *Step 1. Planning the testing:* The elaboration of the test strategy, main objectives, schedule, the level from which the test begins, testing environments, and human resources.
- *Step 2. Requirements testing:* All the specifications used in the design phase are tested by the development team from different points of view: completeness, clarity, consistency, traceability, testability. In case an error is identified, the dysfunctional requirement and associated part of the system architecture are redesigned and integrated within the others.
- *Step 3*. *Testing performed by the development team:* It is structured according to the functional and non-functional features of the RO-SmartAgeing system. It is organized so as to begin with the testing of all the components of the system and their associated functionalities. The next phase is to test the entire system from the point of view of privacy, confidentiality, security, usability, performance, adaptability to a specific environment, and user. The bugs are reported, evaluated, and addressed. The software is updated, and potential dysfunctionalities of the smart environment are solved. Based on the data collected through the smart environment during this step, the development team will test some of the functionalities of the conceptual models of the predictive models.

- *Step 4*. *Testing performed by the users:* All the categories of final users/beneficiaries of the RO-SmartAgeing system are involved in this phase. They have total access to the complete functionalities of the system and the possibility to test it in different environments. The objectives of step 4 are similar to the ones of step 3. Eventual errors or dysfunctionalities are reported, the development team takes them over, and the process is restarted from step 2 or 3.

The successful testing process will end with the implementation of the pilot model of the RO-SmartAgeing system.

In order to organize effective and efficient testing, the following aspects were established and will be used accordingly:

- *Human resources:* The development team involved in testing is divided into three group of testers that simulate the range of users of the RO-SmartAgeing system. Several agreements with public and private healthcare units and associations—“Bucharest Center for Seniors”, ”Nicolae Cajal Association for the Elderly”, “Ana Aslan National Institute of Gerontology and Geriatrics”, “Carol Davila University of Medicine and Pharmacy”, and ”Center for Medical Innovation, InoMed—non-governmental organization”—have been concluded in order to involve representative lots of elderly patients, physicians, and caretakers. Fifty patients (25 women, 25 men) will participate. They have an average age of 78 years, and they are grouped in two lots, according to their Mini-Mental State Examination score: 24 and higher for normal cognition, and 19–23 for MCI. Ten physicians have already agreed to participate, and at least twenty caretakers are going to be involved in testing. The patients and caretakers are recommended by their physicians. They will receive written information, and after their consent, they will sign a consent document in accordance with the legal and ethical provisions in force.
- Locations for testing the RO-SmartAgeing system: Most of the locations in which the RO-SmartAgeing smart environment will be installed are going to be at the elderly patients’ homes. If the local conditions can be assimilated to the ones from a home, the smart environment is scheduled to also be installed inside a nursing home.

## 6. Discussion

The RO-SmartAgeing system aims to support an efficient management of various types of diseases associated with ageing and to promote active and healthy ageing through the development of not only a remote system for healthcare monitoring, but also a complex framework with specific functionalities for new preventative approaches for people with MCI. Important aspects are its capacity to detect any abnormal changes in the MCI-related parameter measurements, as well as the detection of accidental falls. The RO-SmartAgeing system is in line with the current needs and requirements brought about by the ageing of the population, with importance given to the particularities of taking care of patients with MCI and to the stringent necessity of early detection of cognitive decline. Moreover, the system includes the newest trends in implementing remote health-monitoring solutions with the help of age-friendly smart environments.

The research presented in this paper indicated that, taking into consideration the technological and functional characteristics of the RO-SmartAgeing system, the main issues that sustain the benefits that might be obtained through its integration into the health management of an elderly person with MCI can be summarized as follows:

- Cross-patient-centered approaches, which support an efficient management of multimorbidity associated with ageing and MCI, an improved empowerment and responsibility of the patients towards their own health, and an active and healthy ageing, which provides a sustaining framework for health specialists in developing new preventative approaches and therapeutic protocols for people with MCI;
- Implementation of new methods based on scalable ITC services to assess problems related to health, lifestyle, and degenerative diseases related to ageing, and provision of new flexible ways of collecting and processing information and data of a medical nature;
- Development of new AI-based models that integrate high-dimensional data for identification of alarming changes in the cognitive state, behavior, and other vital sign parameters of elderly people with MCI;
- Personalization of services induced by the heterogeneity of the typologies of elderly people with various degenerative dysfunctions and related co-morbidities;
- Substantiation of a new type of long-distance relationship between doctor and elderly person that centralizes several directions of action, such as personalized health monitoring, health status assessment, supporting the independence of the elderly person in a familiar environment, and so on.

Beginning with the design stage of the system, the technology was selected to easily allow both the large range of chosen healthcare functionalities and future upgrading of the systems in terms of software and hardware components. To illustrate this, here are some of the main arguments:

- IoT-based devices provide the potential to provide a continuous stream of real-time health and daily-activities-related data;
- The Withings devices were selected due to their performances, and, no less importantly, to the lack of necessity of having a separate future subscription at the Withings company for total access to the data collected through them (see Supplementary Section S2);
- The smart environment can be personalized not only according to the specific needs of an elderly patient with MCI, but also based on the changes of their health status that are likely to appear in time during the monitoring;
- The trigger alarm functionalities are built in the fog computing in order to get a faster response in case of an abnormal value of a vital sign measurement or behavior being recorded;
- Microservices were chosen due to the fact that they are loosely coupled, organized around a single business capability, and able to be developed with different technologies. These characteristics confer the system fault tolerance, agility, flexibility, scalability, and faster deployment and upgrading;
- As connecting a large number of smart devices increases the risk of vulnerabilities, strict security procedures for applications as well as for the protection of the entire network were developed;
- Current EU standards were used in working with sensitive health data in order to ensure privacy, security, and confidentiality, which will increase confidence in using the system for different types of health specialists, patients, and medical environments;
- The possibility to analyse and process large amounts of data on MCI patient monitoring that were acquired over long periods of time makes it possible to conduct longitudinal studies, and observe meaningful changes in behavior and health for the elderly;
- Recorded data offers the system the opportunity to monitor any problems the MCI patient might encounter in terms of mobility, orientation, self-assessment, memory, or sleep-related or any other cognitive-based activities;
- Mobile networks are the optimal solution to allow real-time access to data collected through the RO-SmartAgeing smart environment. Even if the platform, sensors, and network work well, the accuracy of the diagnosis transmitted to the patient relies on the accuracy of the data provided to the doctor.

Evaluating digital healthcare solutions and technology is a challenging topic, due to their fast rate of emergence, development, and upgrading. Although there is a strong interest in healthcare technology assessment, and an established framework for this purpose exists, there are still drawbacks and limitations that should be tackled and solved in order to be in line with the dynamism of the healthcare domain and the growing empowerment of current patients. The proposed set of evaluation criteria was conceived especially for the specificities of remote health-monitoring systems, in order to provide a trustful framework for an efficient identification of both a good practice and the most appropriate requirements needed for developing a new digital solution.

The main limitation of the RO-SmartAgeing system is that clinical validation has not yet been obtained, since the system is still under testing in a simulated home environment.

## 7. Conclusions

This paper aimed to highlight how a digital solution can efficiently boost an elderly person’s cognitive wellness, health status, and quality of life. It outlined the specific aspects that were tackled in order to develop a remote healthcare system addressing the management of health status of an elderly patient with MCI. The large range of specific functionalities and the MCI-centric approach are novelties in Romania. The target of the RO-SmartAgeing system is to improve the link between healthcare providers and patients; prevent dysfunctions and accidents; avoid institutionalization and hospitalization as much as possible; increase the quality of life of patients in a smart environment, as well as their independence and health; and provide support for the diagnosis and improvement of therapeutic protocols, through the model based on AI for MCI prediction. The scope of the proposed evaluation is to assess the impact of a digital healthcare solution for remote health monitoring so as to provide appropriate knowledge for avoiding and mitigating the negative aspects or consequences, and for highlighting the identified gains, improvements, and successful business models and solutions. The system will be integrated in a hospital information system for a better outpatient management. 

Future research for the RO-SmartAgeing system is anticipated to assimilate some of the measured vital parameters into (bio-)markers as the most predictive for AD. The data will be analysed in a longitudinal manner. 

**Figure 14:**
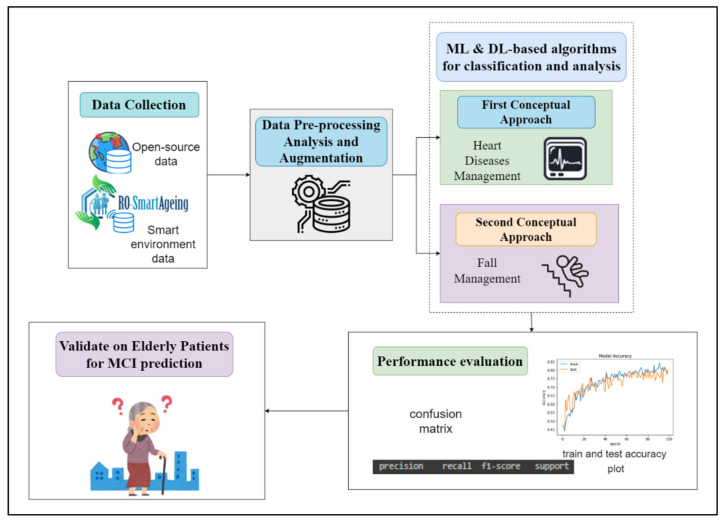
Proposed conceptual application for MCI prediction within RO-SmartAgeing system.

Figure 14 depicts the conceptual application that will be further applied to the RO-SmartAgeing system in order for it to be used for MCI prediction. 

This is an important step for the early detection or prevention of certain MCI-related signs, based on a remote healthcare monitoring system. As the data can be collected through the open-source datasets and RO-SmartAgeing smart environment, a data preprocessing, analysis, and augmentation step is further applied in order to provide the input data to the above-mentioned ML and DL conceptual applications. Based on the output, the evaluation of the performances is obtained in order to decide on the best model to validate on elderly patients for possible MCI prediction.

The future validation of the performance level of the RO-SmartAgeing system will be made for both remote monitoring and for MCI predictions by using clinical/smart environment collected data from elderly patients in real environments, taking into consideration the different physical and cognitive statuses of the monitored persons. Some interactive applications, such as psychometric tests, will also be designed to complete the elderly’s profile. An enhanced predictive model will be further developed in order to improve the early detection of MCI-associated physical degenerations.

## Figures and Tables

**Table 1:** Activity recognition methods [10].

Type of Recognition	Advantages	Necessary Hardware and Software	Limitations
Recognition of simple activities	Collecting data from wearable sensors Applying supervised learning methods [11]	Wearable accelerometers (possibly coupled with biometrical sensors and integrated in clothes)	Contextual information (current location, environmental conditions, ambient objects, etc.) for improving the accuracy of recognition is not considered
	Considering the user’s context	Several environmental sensors	Not suited for more complex activities
Recognition of complex activities	Using sensors to recognize the elderly person’s movements and their interactions with objects and furniture (such as IADLs executed at home)	Time series data analysis method [12] to segment sequences of sensor events. Hidden Markov models inference [13] to recognize activities based on features extracted from recent sensor events. Ontologies to model complex human activities [14]. Machine learning for recognizing activity instances [15], and differentiating cognitively healthy seniors from Alzheimer’s patients based on activity execution and gait events [16].	Detection of abnormal behaviors on a short-term basis Patient’s personal habits are not considered
Long-term analysis of activity data	Model the patient’s usual behavior from the activities performed in the past to detect anomalies as changes	Using an alert system to detect changes in the activity patterns and generate health alerts [17]. Detecting recurrent IADL patterns and their variations by using data mining of heterogeneous multivariate time-series from sensor data [18].	Necessity of big amount of data

**Table 2:** Comparative table of ML and DL performances.

Model		Sensitivity	Specificity	Accuracy
**ML**	**kNN**	87.09%	66.67%	76.01%
	**SVM**	83.87%	63.4%	74%
	**Random Forest**	83.9%	64%	73.8%
	**Logistic Regression**	91%	65%	77%
**DL**		94%	68%	82%

## Data Availability

Not applicable.

## Supplementary Materials

The following supporting information can be downloaded at: https://www.mdpi.com/article/10.3390/healthcare10071214/s1, Supplementary Section S1: technical schemas and final kit for the developed monitoring devices; Supplementary Section S2: devices included in the RO-SmartAgeing system and their corresponding measured parameters and technical characteristics; Supplementary Section S3: Proposed evaluation criteria set for the assessment of health remote monitoring systems for elderly patients, including those with MCI.
