# Supplementary material for: Addressing Mild Cognitive Impairment and Boosting Wellness for the Elderly through Personalized Remote Monitoring
Source: Healthcare (Basel). 2022 Jun 29;10(7):1214. doi: 10.3390/healthcare10071214 (PMC9325232; doi:10.3390/healthcare10071214)
Supplement: Supplementary file 1 [file healthcare-10-01214-s001.zip › Supplementary Section S1_healthcare-1735395.pdf]

Supplementary Section S1 - technical schemas and final kit for the developed monitoring devices

The Medical Box (Figure S1) includes: the integrated sensors; the Arduino MEGA microcontroller board; the ESP8266 Node MCU component, responsible for Wi-Fi data transmission to the Raspberry Pi Gateway.

The sensor interconnection with the Arduino UNO microcontroller board and with the ESP8266 Node MCU component can be visualized in Figure S2.

The Leg Band includes an accelerometer and gyroscope-based sensor for patient’s movements, positioning and fall detection, these measurements being essential for an MCI-related condition (Figure S3).

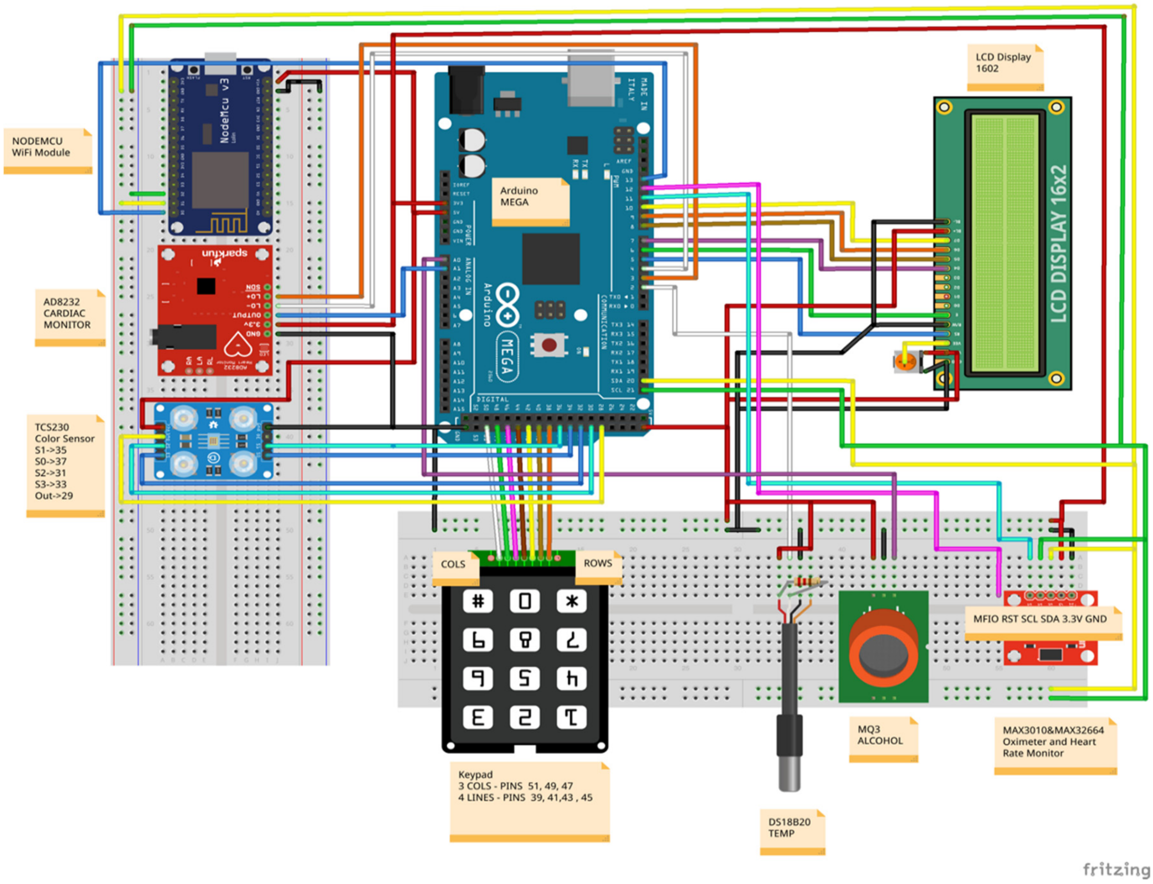

Figure S1. Technical schema of the Medical Blackbox.

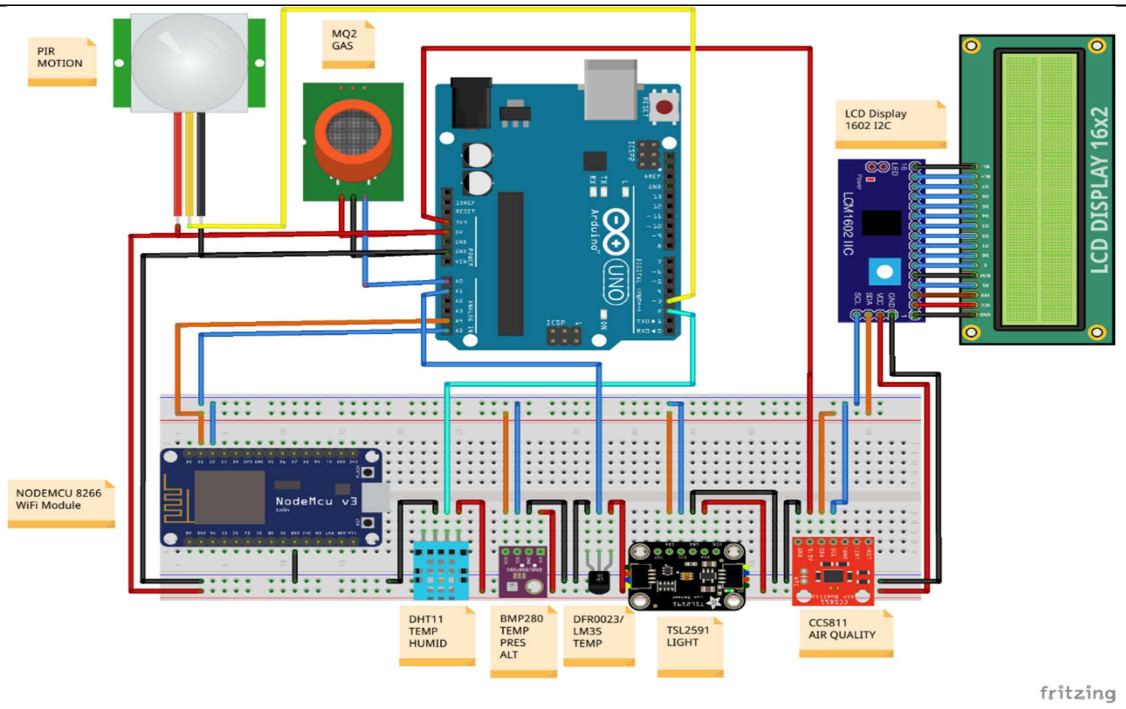

Figure S2. Technical schema of the Ambient Blackbox.

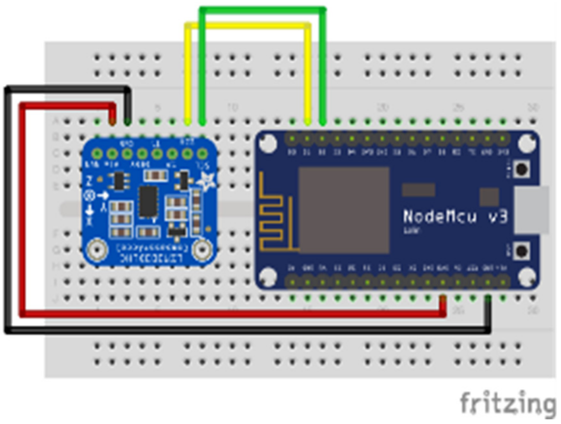

The Leg Band

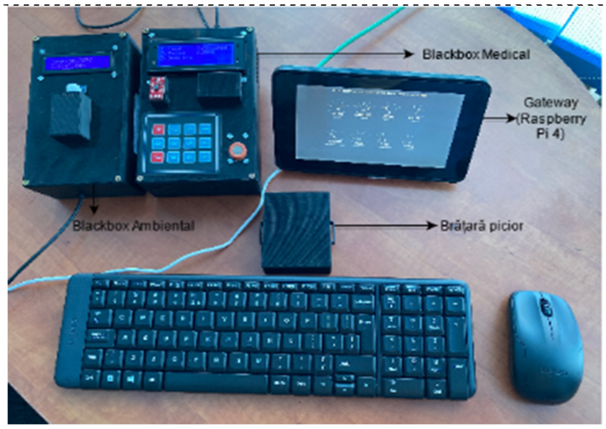

The kit: Medical and Ambient Blackboxes, Leg Band and the Gateway

Figure S3. Technical schema of the Leg Band and the final kit.
