# Supplementary material for: Addressing Mild Cognitive Impairment and Boosting Wellness for the Elderly through Personalized Remote Monitoring
Source: Healthcare (Basel). 2022 Jun 29;10(7):1214. doi: 10.3390/healthcare10071214 (PMC9325232; doi:10.3390/healthcare10071214)
Supplement: Supplementary file 1 [file healthcare-10-01214-s001.zip › Supplementary Section S2_healthcare-1735395.pdf]

Supplementary Section S2 – devices included in the RO-SmartAgeing system and their corresponding measured parameters and technical characteristics

The devices included in the RO-SmartAgeing system and their corresponding targeted measure parameters and technical characteristics are presented in Table S1.

Table S1. RO-SmartAgeing's devices and their targeted measure parameters and descriptions

Device	Targeted measure parameter	Description
**Withings Smartwatch** [1]	ECG<br>Pulse<br>GPS<br>Activity monitoring<br>Score for sleep quality assessment<br>Sleep duration<br>Sleep cycles (deep, light, REM sleep)	The device has a 38 mm diameter and a weight of 32 g which makes it an easy-to-use watch for daily activities. The incorporated sensors used are for ECG, altimeter and accelerometer. The Bluetooth Low Energy syncing with a smartphone makes it a smartwatch which can provide a day and night activity tracking
**Withings Blood Pressure Monitor** [2]	Blood Pressure<br>ECG<br>Pulse	The BPM Core device from Withings is a 3-in1 device which is capable of tracking not only the systolic and diastolic blood pressure, but also the ECG. It also works as a digital stethoscope which is critical for valvular heart disease detection. The data is wirelessly syncing with the smartphone, based on the Health Mate app, and the Withings cloud provides unlimited storage
**Withings Sleep monitor** [3]	Score for sleep quality assessment<br>Breathing disorders<br>Sleep duration, sleep onset and waking time<br>Sleep cycles: deep, light, REM<br>Pulse during sleep<br>Average pulse<br>Snoring duration	The Sleep Analyzer device is capable of real-time recording and computing relevant sleep-related health parameters in order to understand the sleep of the patient (breathing, movement and heart rate) as well as to detect any eventual onset of sleep apnea. As an advanced sleep tracking device, it is able to monitor sleep cycles, provide a sleep score and detect snoring during sleep
**Withings Smart Scale** [4]	Body position detection<br>Bioelectric impedance analysis<br>Body fat (%)<br>Total body water (%)<br>Muscle mass (kg or lb.)<br>Bone mass (kg or lb.)	As it is of great importance to monitor the patient's body-related parameters, the Body+ Smart Scale offers a comprehensive picture of the body composition in order to carefully monitor any changes. The Smart Scale is based on bioelectrical impedance technology as it sends a low-amplitude electrical current through the individual's body, being capable of further measuring the resistance of biological tissues.

Table S.1. *Cont.*

	Device	Targeted measure parameter	Description
**Medical Blackbox**	AD8232 -Single Lead Heart Rate Monitor [5]	ECG	It is used to measure the electrical activity of the heart muscle as it can extract, amplify and filter the small biopotential signals
	Sparkfun Pulse-Oximeter MAX30101&32664 [6]	Pulse and Oxygen in Blood	It incorporates a pulse oximetry sensor and a heart rate module with internal LEDs, photodetectors, optical and electronic elements with an ambient light rejection property. Based on the Cortex M4 processor which is capable of managing all the algorithmic computations, filtering, pressure or position compensation, automatic gain control and R-wave detection, the sensor can almost precisely compute the patient's pulse value as well as oxygen saturation percentage.
	DS18B20 Temperature sensor [7]	Body Temperature	The sensor is based on a 1-Wire interface which needs only one pin for communication and it does not require any external components. It has a measuring range of -55 ° C – +125° C
	MQ3 Alcohol sensor [8]	Volatile Organic Compounds from breath	The sensor is being used to identify the volatile organic compounds concentrations from an individual's breath; The detected volatile compounds are based on alcohol, benzene, methane, hexane, carbon monoxide etc.
	TCS3200 colour detection sensor [9]	Urine biochemistry	The sensor uses urine strips to detect relevant parameters of urine biochemistry, based on RGB LEDs
**Leg Band** - Grove – 6-axis Accelerometer and Gyroscope [10]		Fall Detection & No. of steps	Based on a 6-axis accelerometer and gyroscope, the Leg Band can identify acceleration of body changes and body rotation speed. The technical specifications include 5V of Input voltage, an I2C interface and 2 independent programmable interrupt generators.

Table S.1. *Cont.*

	Device	Targeted measure parameter	Description
**Ambient Blackbox**	DRF0023 Temperature sensor [11]	Ambient Temperature	The temperature sensor is based on a functional detection range of 0-100°C and a sensitivity of 10 mV per degree Celsius. The operating voltage is 3.3-5 V and it has an analog interface.
	DHT11 Temperature and humidity sensor [12]	Environmental Temperature and Humidity	The temperature and humidity sensor can measure humidity and temperature in the range of 20-90% and 0-50°C, respectively.
	Light sensor TSL2591 [13]	Ambient Light	The light sensor can detect ambient light in a range between 188 μLux - 88000 Lux. It contains both infrared and full spectrum diodes (this means that infrared light, full spectrum or visible spectrum can be measured separately);
	MQ2 Gas sensor [14]	Flammable gases and smoke	The gas sensor can identify combustible gas and smoke. The output voltage from the Gas sensor expands with the concentration of gas. The potentiometer can be rotated in order to adjust the sensitivity.
	Grove – PIR Motion sensor [15]	Motion	The Grove-PIR Motion sensor can detect the infrared signal caused by motion: if the PIR sensor detects infrared energy, the motion detector is triggered and the sensor has a HIGH output on the SIG pin.
	BMP280 Pressure sensor [16]	Pressure and altitude	The pressure changes with the altitude and the pressure measurements are accurate with the BMP280 sensor incorporated in the Ambient Blackbox. It has a supply voltage of 3-6V and an I2C communication interface.
	CCS811 Air Quality sensor [17]	Air Quality	The CCS811 Air Quality sensor features a metal oxide gas sensor that can detect a wide range of volatile compounds. It has a microcontroller unit that incorporates an analog-to-digital converter and an I2C communication interface.

Table S.1. *Cont.*

Device	Targeted measure parameter	Description
**Smart depth camera (Orbbec Persee)** [18]	Human activity recognition	Orbbec Persee represents a powerful smart depth camera with a built-in ARM processor, RGB and depth sensors. It has 8GB of internal storage that can be expanded with a MicroSD card, 2GB of DDR3 RAM, enough field of view for monitoring applications and can film up to a distance of 8 m.

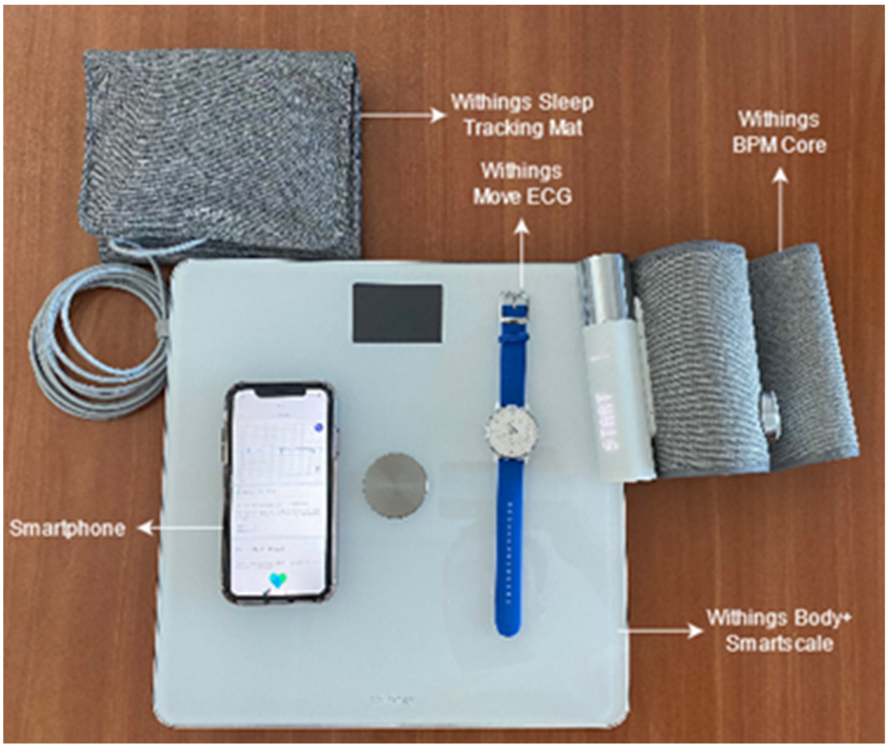

Figure S4. *Withings devices*

A picture of the Withings devices is provided in Figure S4. The most relevant measured parameters and their corresponding reference values that are taken into consideration to compare the measured parameters in order to generate potential trigger alarms, are presented in the following table (Table S2).

Table S2. The most relevant measured parameters and their corresponding reference values.

Measured Parameter	Normal and reference values
Heart rate	55-80 beats/minute
Oxygen Saturation	>95%
Temperature	Normal: 36.1-37 °C<br>Low fever: 37.1-37.3 °C<br>Mild fever: >37.4-38 °C<br>Fever: 38.1-39 °C<br>High fever: 39.1-40.4 °C<br>Hyperpyrexia: ≥40.5 °C

Table S2. *Cont.*

Measured Parameter	Normal and reference values
Sleep duration	6-8 hours
Blood pressure	Low: <100/60 mmHg<br>Normal: 100 / 60-130 / 80 mmHg<br>Slightly increased: 130 / 80-140 / 90 mmHg<br>Increased: >140/90 mmHg<br>Hypertensive crisis: >180/100 mmHg
Score for sleep quality assessment	>75 – high; 50-75 – medium; <49 – low
Body fat (%)	Women: 25-35%<br>Men: 15-25%
Total body water (%)	50-60%
Muscle mass (kg or lb.)	15-20%
Bone mass (kg or lb.)	8-10%
Normal blood sugar for diabetics	On an empty stomach: <100 mg / dl
	Before meals: 70-130 mg / dl
	After a meal (1-2 hours): <180 mg / dl
	Before movement: >100 mg / dl
	Before bedtime: 100-140 mg / dl
Normal blood glucose	60-99 mg / dl
Modified basal glycemia (pre-diabetes)	100-125 mg / dl
Diabetes	>126 mg / dl

References

1. Record an ECH anytime, anywhere. Available online: https://www.withings.com/de/en/move-ecg (accessed on 7 February 2022).
2. Manage your blood pressure & detect silent heart conditions. Available online: https://www.withings.com/de/en/bpm-core (accessed on 7 February 2022).
3. Sleep-lab results, at home. Available online: https://www.withings.com/de/en/sleep-analyzer (accessed on 8 February 2022).
4. Knowlegde is power. Available online: https://www.withings.com/it/en/scales (accessed on 8 February 2022).
5. Interfacing AD8232 Heart Rate Monitor – ECG Sensor Module with Arduino. Available online: https://electropeak.com/learn/interfacing-ad8232-heart-rate-monitor-ecg-sensor-module-with-arduino/ (accessed on 10 February 2022).
6. SparkFun Pulse Oximeter and Heart Rate Sensor - MAX30101 & MAX32664. Available online: https://www.sparkfun.com/products/15219 (accessed on 10 February 2022).
7. DS18B20 Programmable Resolution 1-Wire Digital Thermometer. Available online: https://datasheets.maximintegrated.com/en/ds/DS18B20.pdf (accessed on 10 February 2022).
8. Technical Data MQ-3 Gas Sensor. Available online: https://www.sparkfun.com/datasheets/Sensors/MQ-3.pdf (accessed on 11 February 2022).
9. TCS3200 Color Sensor Module. Available online: https://components101.com/sensors/tcs3200-color-sensor-module (accessed on 11 February 2022).
10. Grove - 6-Axis Accelerometer & Gyroscope. Available online: https://www.seeedstudio.com/Grove-6-Axis-Accelerometer-Gyroscope.html (accessed on 11 February 2022).
11. DFRobot LM35 Linear Temperature Sensor. Available online: https://www.digikey.com/htmldatasheets/production/2061108/0/0/1/dfr0023.html (accessed on 11 February 2022).
12. DHT11–Temperature and Humidity Sensor. Available online: https://components101.com/sensors/dht11-temperature-sensor (accessed on 11 February 2022).
13. Adafruit TSL2591 High Dynamic Range Digital Light Sensor. Available online: https://www.adafruit.com/product/1980 (accessed on 12 February 2022).
14. MQ2 Gas Sensor. Available online: https://components101.com/sensors/mq2-gas-sensor (accessed on 12 February 2022).
15. Grove - PIR Motion Sensor. Available online: https://wiki.seeedstudio.com/Grove-PIR_Motion_Sensor/ (accessed on 12 February 2022).

16. BMP280 Barometric Pressure Sensor Module. Available online: https://www.optimusdigital.ro/en/pressure-sensors/1666-modul-senzor-de-presiune-barometric-bmp280.html (accessed on 12 February 2022).
17. Adafruit CCS811 Air Quality Sensor. Available online: https://learn.adafruit.com/adafruit-ccs811-air-quality-sensor (accessed on 12 February 2022).
18. Persee. Available online: https://orbbec3d.com/index/Product/info.html?cate=38&id=37 (accessed on 2 May 2022)
