# Supplementary material for: Addressing Mild Cognitive Impairment and Boosting Wellness for the Elderly through Personalized Remote Monitoring
Source: Healthcare (Basel). 2022 Jun 29;10(7):1214. doi: 10.3390/healthcare10071214 (PMC9325232; doi:10.3390/healthcare10071214)
Supplement: Supplementary file 1 [file healthcare-10-01214-s001.zip › Supplementary Section S3-healthcare-1735395.pdf]

Supplementary Section S3 - Proposed evaluation criteria set for the assessment of health remote monitoring systems for elderly patients, including those with MCI

Table S3. Proposed evaluation criteria set for the assessment of health remote monitoring systems for elderly patients, including those with MCI.

Criteria	Sub-criteria	Layers *				
		PL	CL	E/FL	CL	A&VL
	**Module 0: General**					
Type of beneficiaries	elderly patients	√				√
	patients with MCI	√				√
	caretakers	√				√
	physicians	√				√
	health specialists	√				√
	healthcare units					√
Basic characteristics of beneficiaries	health literacy	√				√
	IT literacy	√				√
	experience with remote health monitoring technologies	√				√
	demands					√
Level of adoption of the system	experimental model	√	√	√	√	√
	tested and deployed	√	√	√	√	√
	integrated in restricted medical practice	√	√	√	√	√
	fully integrated and implemented within a business model	√	√	√	√	√
	upgraded	√	√	√	√	√
Regulation and legislation compliance	General Data Protection Regulation (GDPR)- EU			√	√	√
	Health Insurance Portability and Accountability Act (HIPAA) – USA			√	√	√
	other regulatory measures			√	√	√
Responsibility	specialists’office & clinic	√	√			√
	clinic	√	√			√
	hospital	√	√			√
	nursing home & assisted living facilities	√	√			√
	outpatient clinic	√	√			√
	foundation & association	√	√			√
	**Module 1: Parameter Acquisition**					
Type of devices	wearable sensors	√	√			
	stationary sensors	√	√			
	RFID	√	√			
	actuators	√	√			
	smart sensors	√	√			
	video cameras	√	√			
	other	√	√			
Characteristics of acquisition devices	integrated sensors	√				
	Wi-Fi transmission	√	√			
	low power consumption	√				

Table S3. *Cont.*

Criteria	Sub-criteria	Layers *				
		PL	CL	E/FL	CL	A&VL
Connectivity	smartphones	√	√			√
	tablets/personal computers	√	√			√
	Wi-Fi connectivity		√			
	other		√			
	interference management		√			
**Module 2: Analysis**						
Data	intuitive					√
	informative					√
	accurate			√	√	√
	complete			√	√	√
	validated			√	√	√
	available			√	√	√
	normative compliant			√	√	√
	properly identified[1]			√	√	√
Decision support	computational power			√	√	
	localized data analytic capacities			√	√	
	predictive capacities			√	√	
	real-time responsiveness to emergencies			√	√	
	high-accuracy & precision of algorithms & methods			√	√	
Computation of	vital signs			√	√	
	activity patterns			√	√	
	ambient factors			√	√	
	movement elements			√	√	
	other			√	√	
Storage	Proper storage mechanisms[2] for:			√	√	
	- structured data			√	√	
	-unstructured data			√	√	
Infrastructure for high-level capabilities	effective and sustainable				√	
	scalable				√	
	multi-cloud integrability				√	
	back-up features				√	
Management	data management			√	√	
	device management			√	√	
	build-deploy-upgrade management			√	√	
**Module 3: Application & Business**						
Patient profile	interactive access to personal data	√				√
	update personal health data	√				√
	adequate visualization mechanisms					√

[1] Data should be uniquely identified, without any possibility of being misinterpreted or duplicated

[2] Proper storage mechanisms should ensure features like: high availability and accessibility, reliability of data and service, efficient data management, flexibility and scalability, maintainability of the health database.

Table S3. *Cont.*

Criteria	Sub-criteria	Layers *				
		PL	CL	E/FL	CL	A&VL
Health status management	chronic disease				√	√
	elderly care				√	√
	MCI care				√	√
	medication care				√	√
	daily activities				√	√
	other				√	√
Impact on beneficiaries	patient satisfaction					√
	caretaker satisfaction					√
	physician satisfaction					√
	health specialist satisfaction					√
	improved health status:					√
	-clinical					√
	-cognitive					√
	-behavioural					√
	- well-being					√
	- independence					√
	decreased readmissions, emergency situations and institutionalizations					√
	improved patient-physician interaction					√
	lower adverse reactions					√
	controlled risk factors					√
	lower mortality					√
	lower morbidity					√
	equity					√
**Module 4: Technical Features**						
Most used technology	Cloud computing			√	√	
	Edge / Fog computing			√	√	
	IoT	√		√	√	
	AI			√	√	
	Big Data & Big Data Analytics			√	√	
	mobile			√	√	
	others		√	√	√	
Content quality	accuracy			√	√	√
	relevance			√	√	√
	consistency			√	√	√
	completeness			√	√	√
	timeliness			√	√	√
	volume			√	√	√
Tailorability	user-centered	√		√	√	√
	Customizable	√		√	√	√
	personalizable	√		√	√	√
	flexibility	√		√	√	√

Table S3. *Cont.*

Criteria	Sub-criteria	Layers *				
		PL	CL	E/FL	CL	A&VL
Usability	understandability	√				√
	memorability / learnability	√				√
	efficiency	√				√
	satisfaction	√				√
	recoverability	√			√	√
	feedback					√
	age-friendliness	√				√
Accessibility	remote access	√	√			√
	response time/responsiveness		√		√	√
	improved access to larger range of data		√		√	√
Interoperability	use of open standards		√	√	√	
	compatibility with other digital health solutions	√	√	√	√	
	integration with other digital health solutions	√	√	√	√	
Privacy & Security & Confidentiality	data protection	√	√	√	√	√
	data security	√	√	√	√	√
	data confidentiality	√	√	√	√	√
	device security & protection	√	√	√	√	
	access control	√	√	√	√	√
	privacy-by-design	√	√	√	√	√
	trust	√	√	√	√	√
Feasibility	functional	√	√	√	√	√
	technical	√	√	√	√	√
	operational	√	√	√	√	√
	financial	√	√	√	√	√
Characteristics of the life cycle of solution	scalability	√	√	√	√	√
	flexibility	√	√	√	√	√
	agility	√	√	√	√	√
	composability	√	√	√	√	
	low latency	√	√	√		
	technology heterogeneity	√	√	√	√	√
	exploitation	√		√	√	√
	independently deployable	√	√	√	√	√
	independently running components around business capabilities			√	√	√
	decentralized data management				√	
	fault tolerance	√		√	√	√
	exploitation	√	√	√	√	√
	support & maintenance	√	√	√	√	√
**Module 5: Available Evidence on Outcomes**						
Features of provided services	preventative					√
	proactive					√
	reactive					√
	personalized	√			√	√
	continuous	√	√		√	
	patient-centered	√			√	√

Table S3. *Cont.*

Criteria	Sub-criteria	Layers *				
		**PL**	**CL**	**E/FL**	**CL**	**A&VL**
Innovative features of healthcare services	improved access to personal health data					√
	improved access to healthcare services					√
	improved quality of healthcare management					√
	increased patient empowerment in their own health status management					√
	increased patient awareness regarding healthcare					√
Cost effectiveness	reduced direct health practice costs					√
	smart environment acquisition	√				
	unit per patient per year	√				
	set up & training	√				√
	improved use of human services					
	staff cost	√				√
Customer support	24/7 support	√				√
	availability on phone	√				√
	online assistance	√				√
	training	√				√
	maintenance	√				√

* PL - Perception Layer, CL - Communication Layer, E/FL - Edge/Fog Layer, CL - Cloud Layer, A&VL -Application & Visualization Layer, √ - associated
